# Targeting ErbB2 and ErbB3 with a bispecific single-chain Fv enhances targeting selectivity and induces a therapeutic effect *in vitro*

**DOI:** 10.1038/sj.bjc.6604700

**Published:** 2008-10-07

**Authors:** M K Robinson, K M Hodge, E Horak, Å L Sundberg, M Russeva, C C Shaller, M von Mehren, I Shchaveleva, H H Simmons, J D Marks, G P Adams

**Affiliations:** 1 Department of Medical Oncology, Fox Chase Cancer Center, Philadelphia, PA, USA; 2 Department of Anesthesia and Pharmaceutical Chemistry, University of California, San Francisco, CA, USA

**Keywords:** engineered antibody, bispecific, ErbB

## Abstract

Inappropriate signalling through the EGFR and ErbB2/HER2 members of the epidermal growth factor family of receptor tyrosine kinases is well recognised as being causally linked to a variety of cancers. Consequently, monoclonal antibodies specific for these receptors have become increasingly important components of effective treatment strategies for cancer. Increasing evidence suggests that ErbB3 plays a critical role in cancer progression and resistance to therapy. We hypothesised that co-targeting the preferred ErbB2/ErbB3 heterodimer with a bispecific single-chain Fv (bs-scFv) antibody would promote increased targeting selectivity over antibodies specific for a single tumour-associated antigen (TAA). In addition, we hypothesised that targeting this important heterodimer could induce a therapeutic effect. Here, we describe the construction and evaluation of the A5-linker-ML3.9 bs-scFv (ALM), an anti-ErbB3/ErbB2 bs-scFv. The A5-linker-ML3.9 bs-scFv exhibits selective targeting of tumour cells *in vitro* and *in vivo* that co-express the two target antigens over tumour cells that express only one target antigen or normal cells that express low levels of both antigens. The A5-linker-ML3.9 bs-scFv also exhibits significantly greater *in vivo* targeting of ErbB2‘+’/ErbB3‘+’ tumours than derivative molecules that contain only one functional arm targeting ErbB2 or ErbB3. Binding of ALM to ErbB2‘+’/ErbB3‘+’ cells mediates inhibition of tumour cell growth *in vitro* by effectively targeting the therapeutic anti-ErbB3 A5 scFv. This suggests both that ALM could provide the basis for an effective therapeutic agent and that engineered antibodies selected to co-target critical functional pairs of TAAs can enhance the targeting specificity and efficacy of antibody-based cancer therapeutics.

Although many treatment options exist for metastatic breast cancer, additional curative strategies are required. The development of antibodies that target critical signalling pathways implicated in cancer initiation and progression is one area of active research focused on this concern. The ErbB family (ErbB1/EGFR, ErbB2/HER2, ErbB3/HER3 and ErbB4/HER4) of receptor tyrosine kinases (RTKs) is known to drive both formation and progression of a number of commonly occurring cancers, including breast cancer, due to the normal role of these RTKs in regulating cell growth and survival (Yarden and Sliwkowski, 2001; Hynes and Lane, 2005). Therefore, these RTKs are important targets for the development of new antibody-based therapeutics. At present, the anti-ErbB2 monoclonal antibody (MAb) trastuzumab is the only antibody approved for use against metastatic breast cancer. Trastuzumab, most likely due to a multifaceted mechanism (for review, see Hynes and Lane, 2005) is efficacious against cancer that is driven by ERBB2 gene amplification (Slamon *et al*, 2001). This subset of cancer is associated with poor patient outcome due to enhanced tumour aggressiveness and high risk of relapse (Slamon *et al*, 1989) but accounts for only 20–30% of the total patient population. Increased patient coverage is necessary. A second anti-ErbB2 MAb, pertuzumab, binds to an epitope in domain II of the extracellular domain (ECD) sterically inhibiting the binding pocket necessary for receptor dimerisation (Hynes and Lane, 2005). By preventing dimerisation, pertuzumab effectively blocks ligand-dependent proliferation of non-gene-amplified breast and prostate cancer cells in culture (Agus *et al*, 2002). These results have led to the initiation of clinical trials in non-gene-amplified patient populations, although with limited success to date (Gordon *et al*, 2006; Walshe *et al*, 2006; Agus *et al*, 2007; de Bono *et al*, 2007).

In contrast to eliciting a therapeutic effect by directly altering signalling events, antibodies can be used to selectively deliver cytotoxic payloads to cancer cells (for review, see Adams and Weiner, 2005). However, tumour-associated antigens are often expressed at lower levels on normal tissues and are known to function as ‘off-site’ targets for therapeutic antibodies. For example, acneform rash associated with cetuximab-dependent inhibition of EGFR signalling in the skin is correlated with clinical responses in colorectal cancer patients (Cunningham *et al*, 2004). In some cases, targeting an antigen present in normal tissues can lead to significant toxicity or even death, particularly when a cytotoxic immunoconjugate is employed (Pai *et al*, 1991; Knox *et al*, 2000; Seidman *et al*, 2002). One possible mechanism to enhance the therapeutic efficacy of MAb-based treatment is to improve the targeting selectivity of these agents. Tumour targeting of small, engineered antibodies is greatly influenced by the functional affinity imparted through avid binding (Adams *et al*, 1993, 2006). We hypothesised that this contribution of functional affinity could be exploited, through the use of bispecific antibodies (bsAbs), to improve tumour uptake and targeting selectivity over normal tissue that expresses only one (or low levels of both) target antigen. Other groups have developed bsAb-based molecules that simultaneously target two tumour-associated antigens such as EGFR and insulin growth factor receptor (IGFR) (Lu *et al*, 2005) or carcinoembryonic antigen (CEA) and ErbB2 (Dorvillius *et al*, 2002). However, there has been little evidence that bsAb-based targeting strategies can be employed to enhance targeting specificity.

On the basis of multiple lines of evidence, we hypothesised that the ErbB2/ErbB3 heterodimer would be a valuable target for the development of a bsAb for the treatment of breast cancer. First, the ErbB2/ErbB3 heterodimer constitutes the most potent ErbB signalling pair when measured in cell culture assays (Yarden and Sliwkowski, 2001; Hynes and Lane, 2005). Second, expression of either ErbB2 or ErbB3 is associated with poor prognosis in breast cancer. Third, the receptors are frequently co-expressed in human breast cancer (Gasparini *et al*, 1994; Naidu *et al*, 1998; Witton *et al*, 2003); Naidu *et al* (1998), in a 220 patient study, showed that greater than 50% of all tumours expressed moderate to high levels of either ErbB2 or ErbB3, with their co-expression being higher than any other pair of ErbB family members. To that end, we developed an anti-ErbB2/ErbB3 bispecific single-chain Fv molecule. Here, we present both *in vitro* and *in vivo* evidence of its selective targeting of cells that co-express both target antigens. In addition, we demonstrate that the bispecific single chain-Fv (bs-scFv) has intrinsic anti-cancer activity when measured *in vitro* and that the anti-ErbB3 arm of the bs-scFv is responsible for mediating this activity.

## Materials and methods

### Cell lines

The BT-474 (ATCC no. HTB-20), SK-OV-3 (ATCC no. HTB-77), MDA-MB-468 (ATCC no. HTB-132), MCF10a (ATCC no. CRL-10317) and B16-F0 (ATCC no. CRL-6233) cell lines were obtained from the American Type Culture Collection (ATCC). MDA-361/DYT2 is a derivative of MDA-MB-361 (ATCC no. HTB-27) and was a kind gift from Dr D Yang (Georgetown University; Yang *et al*, 1998). All cells were maintained in DMEM supplemented with 10% fetal bovine serum (FBS). MVM2 cells are a derivative of B16-F0 murine melanoma cells transfected to express full-length human ErbB2 under control of the CMV promoter. The human cDNA of ErbB2 was obtained from pSVHER2 (gift from Dr T Eberlein; Yoshino *et al*, 1994) and subcloned into pLNCX as a 4.6 Kb *Hin*dIII fragment to create pLNCX-HER2. pLNCX-ERBB2 was transfected into B16-F0 cells through electroporation, and transfectants were selected in DMEM supplemented with 10% FBS and 1.8 mg ml^−1^ G418. Clones were expanded and cell surface expression of ErbB2 was confirmed by flow cytometry with mouse anti-ErbB2 MAb 520C9 (gift from Dr D Ring, Chiron Corporation) and goat anti-mouse-FITC conjugated secondary antibody. The MVM2 clone was selected on the basis of its high level of expression by flow cytometry. Expression levels of ErbB2 and ErbB3 on each of the cell lines used in this study were measured by quantitative flow cytometry with the QuantiBrite System (Becton Dickinson) using the manufacturer's protocols. Anti-ErbB2 (Becton Dickinson, cat no. 340552) and anti-ErbB3 (R&D, cat no. FAB3481P) conjugated to a 1 : 1 ratio with phycoerythrin (PE) by the manufacturers were used for monitoring expression levels with the mean of three independent experiments reported in Table 1.

### Construction of bs-scFv

The anti-ErbB3/anti-ErbB2 bs-scFv was created from the A5 anti-ErbB3 (Horak *et al*, 2005) and ML3.9 anti-ErbB2 (Schier *et al*, 1996) scFvs using a multistep cloning strategy. Briefly, sense (5′<AATTCAGGTGCTGGTACTTCAGGTTCAGGTGCTTC AGGTGAAGGTTCAGGTTCAA>3′) and anti-sense (5′<AGCTTTGAACCTGAACCTTCACCTGATGCACCTGA ACCTGAAGTACCAGCACCTG>3′) oligos encoding a peptide linker sequence (NSGAGTSGSGASGEGSGSKL) were hybridised and cloned into pET20b(+) (Novagen) as an *Eco*RI/*Hin*dIII fragment to create pET20b-L. The anti-ErbB3 scFv was amplified from pSYN-A5 (Horak *et al*, 2005) by PCR with primers that encompassed the *Nco*I site at the 5′-end of the gene and incorporated an in-frame *Eco*RI site at the 3′-end of the gene. The fragment was subcloned into pET20b-L as an *Nco*I/*Eco*RI fragment to create pET20b-AL. The ML3.9 scFv was amplified from pSYN-ML3.9 (Schier *et al*, 1996) with primers that incorporated in-frame *Hin*dIII and *Xho*I sites at the 5′- and 3′-ends of the gene, respectively, and was then subcloned into pET20b-AL as a *Hin*dIII/*Xho*I fragment to create pET20b-A5-linker-ML3.9 bs-scFv (ALM). The A5-linker-ML3.9 bs-scFv was then subcloned into pSYN as an *Nco*I/*Not*I fragment. This construct (pSYN-ALM) contains an *Xho*I site, 6XHIS tag and stop codon derived from the pET20b(+) vector 5′ to the pSYN-encoded *Not*I site. To facilitate substitution of individual arms, the *Eco*RI and *Hin*dIII sites present in the polylinker of pSYN-ALM were destroyed by site-directed mutagenesis with the QuikChange Site-Directed Mutagenesis kit (Stratagene) using the manufacturer's protocols.

The ‘dead’ D3 scFv was isolated from a human phage-display library (Yuan *et al*, 2006) and binds to an epitope formed by the junction between a biotinylated peptide and streptavidin. Binding, as measured by surface plasmon resonance (SPR) on a BIAcore1000 (BIAcore, Piscataway, NJ, USA), requires the presence of both the peptide and SA; binding cannot be competed with either of the components alone. The D3 scFv was amplified by PCR with primers that incorporate either in-frame *Nco*I/*Eco*RI or *Hin*dIII/*Xho*I restriction sites and sublconed into pSYN-ALM to create pSYN-DLM and pSYN-ALD.

### Physical characterization of bs-scFvs

Bispecific-scFvs were expressed in *Escherichia coli* and purified by sequential IMAC and size-exclusion chromatography essentially as described (Robinson *et al*, 2005). Binding of the A5 and the ALM bs-scFv to ErbB2 and ErbB3 ECDs was characterized by SPR using ErbB2 and ErbB3 ECDs (Horak *et al*, 2005) as target antigens and methods described previously (Yuan *et al*, 2006). Extracellular domains were diluted to 10 *μ*g ml^−1^ in 10 mM sodium acetate, pH 5.2, and approximately 200 RU of ECDs were immobilized onto CM5 sensor chips through NHS-ester chemistry. Kinetic constants for A5 were determined by passing serially diluted samples (0 nM to 2 *μ*M) over flow cells at a flow rate of 40 *μ*l min^−1^. Response against an ErbB2-coated flow cell was used as a negative control and subtracted from the response generated against the ErbB3 ECD to obtain the final sensorgrams. Data was evaluated using BIAEvaluation 3.2 software (BIAcore, Piscataway, NJ, USA) and fit using the 1 : 1 Langmuir binding model. Flow cells were regenerated by sequential 15 s pulses with 10 mM glycine, pH 2, and 50 mM triethylamine, pH 10, followed by equilibration with PBS running buffer.

Stability of ALM in human serum was assayed as follows. The A5-linker-ML3.9 bs-scFv was radioiodinated with Iodine-125 (Perkin Elmer, cat no. NEZ033H) using iodogen-coated glass tubes (Pierce, cat no. 28601) essentially as described (Fraker and Speck, 1978). Briefly, 300–500 *μ*g of a bs-scFv was labelled with 0.75–1.35 mCi of Na^125^I (17 Ci mg^−1^, 350 mCi ml^−1^) containing 10 *μ*M carrier NaI. Labelled protein was purified from non-incorporated ^125^I by size-exclusion chromatography over a PD10 column (Amersham Pharmacia) and percentage of incorporation was determined by ITLC (Biodex). This protocol resulted in radioiodination of the bs-scFvs at efficiencies between 14 and 42% and produced product with a specific activity of 0.5–1.3 *μ*Ci *μ*g^−1^ that was >95% pure by ITLC. Immunoreactivity of the radiolabelled bs-scFvs ranged from being 50 to 67% active as judged by live-cell binding assays with SK-OV-3 cells as described previously (Adams *et al*, 1993). Radioiodinated ALM (200 ng *μ*l^−1^) was diluted 10-fold in human serum (final volume 100 *μ*l) and incubated at 37°C over a time course of 72 h. At each time point, a 10 *μ*l aliquot was removed and retention of activity, as compared with *T*=0, was determined by live-cell binding. The FDA-approved anti-ErbB2 MAb trastuzumab was radioiodinated and assayed side by side as a positive control.

### *In vitro* selectivity

MDA-MB-468 cells (ErbB2−/ErbB3+) were stained with 20 *μ*M Cell Tracker Blue CMAC (Molecular Probes, cat no. C-2110), MVM2 (ErbB2+/ErbB3−) and MCF10a (ErbB2+/ErbB3+) cells were stained with 10 *μ*M Cell Tracker Orange CMTMR (Molecular Probes, no. C-2927) and BT-474 cells (ErbB2+/ErbB3+) were left unstained, as appropriate for each individual experiment. Cells were plated in six-well plates (300 000 cells per well) and allowed to adhere overnight. Once adhered, cells were incubated in serum-free DMEM containing appropriate Cell Tracker dyes for 45 min, wells were rinsed with Dulbecco's PBS (Invitrogen, cat. no. 14287072) to remove excess dye and cells were allowed to recover in DMEM containing 10% FBS for 30 min. Following recovery, cells were trypsinized and counted. To determine the effect of bivalent *vs* monovalent targeting, cells were mixed in equal ratios, incubated with ALM at concentrations of 1 *μ*M, 0.1 *μ*M, 10 nM, 1 nM or 100 pM for 30 min at 4°C, washed with PBS and bound ALM was detected through its 6XHis tag with an anti-PentaHis-Alexa Fluor 488 MAb (Qiagen, cat. no. 35310). The samples were subjected to flow cytometric analysis using either a FACS-LSIIR equipped with two lasers or a FACS-VantageSE/DiVa machine (Becton Dickinson) equipped with three lasers, one UV-capable, as necessary. The forward *vs* side-scatter parameters were similar for all the cell lines allowing analysis as a single population. The Blue cell tracker dye was detected at the Cascade blue channel and the Orange Cell tracker dye at the PE channel. Three-color flow cytometry data were acquired using CELLQuest software (Becton Dickinson) and then analysed using the FlowJo software package (Tree Star Inc.). Data are representative of three independent experiments of 9 × 10^5^cells per sample with ⩾50 000 events collected. To determine the effect of low-level expression of both ErbB2 and ErbB3 on ALM targeting, an increasing number of MCF10a cells were mixed with 2 × 10^5^ BT-474 cells at ratios of 1 : 1 to 18 : 1, incubated with ALM (100 nM) and analysed by flow cytometry (1 × 10^5^ events were collected) as described above.

### Biodistribution

Male CB.17 ICR *scid* mice, 6–8 weeks of age, were obtained from the Fox Chase Cancer Center Laboratory Animal Facility, and cells were implanted as follows. SK-OV-3 (3.0 × 10^6^), MDA-MB-468 (6.0 × 10^6^) or MVM-2 (3 × 10^6^) were implanted subcutaneously into the inguinal region of the mice. BT-474 (3.0 × 10^6^) cells were implanted following subcutaneous implantation of 17-*β*-estradiol pellets (Innovative Research of America, cat. no. SE-121). At 3–7 weeks post-implantation, depending on cell line, tumours were 100–300 mm^3^ in size. Lugol's solution (Sigma, cat. no. L6146) was added to the drinking water at a final concentration of 0.2% to block thyroid accumulation of radioiodine, and biodistribution studies were initiated approximately 5 days later. Bispecific single chain FVS were radioiodinated as described above, and ^125^I-bs-scFvs (∼20 *μ*g per mouse) were administered to cohorts of mice (*n*⩾5 per cohort) through tail-vein injection, mice were euthanized at indicated times post-injection and dissected, and major organs were weighed and counted in a *γ* well counter (Cobra Quantum, Packard Instruments) with a window of 15–75 keV. The retention in tumour and non-target tissues was expressed as a percentage of the injected dose localised per gram of tissue (% ID per g) as determined from decay-corrected counts as described previously (Adams *et al*, 1993). The mean and standard error of the mean (s.e.m.) values reported are typical results obtained from at least two replicates of each experimental condition.

### *In vitro* efficacy

MDA-361/DYT2 and BT-474 cells (1.5 × 10^5^) were plated in six-well plates (Nunclon, cat. no. 140685) and allowed to adhere overnight. Cells were left untreated, treated with increasing concentrations of ALM for 4 days or treated with 4 *μ*M doxorubicin (Pharmacia & Upjohn) for 24 h. Cells were then analysed for apoptosis by flow cytometry with the Guava Nexin kit using the manufacturers protocol. Samples were analysed using a Guava Technologies Personal cytometer with Annexin V-PE and the membrane impermeant dye 7-Amino-actinomycin D (7-AAD) as markers for early and late apoptosis, respectively. Cell cycle compartment analysis was performed by flow cytometry. Cells (1 × 10^6^) were fixed with ice-cold 70% ethanol, treated with RNaseA (1 mg ml^−1^) for 30 min and then stained with propidium iodine at a concentration of 50 mg ml^−1^. Cells were analysed by flow cytometry on a Becton Dickinson FACscan flow cytometer with CellQuest DNA software. Inhibition of colony formation was performed as follows. MDA-361/DYT2 and BT-474 were seeded into six-well plates (6000 cells per well) in a total volume of 2 ml media and allowed to adhere overnight. In triplicate, media containing the appropriate antibodies (as described in figure legends) were added to cells and then refreshed every 5 days. After 2 weeks, colonies were fixed in 70% ethanol and then stained with 0.25% methylene blue and 30% ethanol, and colonies were counted with an automatic colony counter (MiniCount, IPI Inc.) with a cutoff of 0.35 mm. Data presented for all *in vitro* analyses represent typical results from at least three independent experiments. Pertuzumab (hu2C4, Genentech) at a concentration of 300 nM was used as a positive control for these experiments.

### Statistical analysis

Average and s.e.m. were calculated for all organs and tumours in the biodistribution experiments. Averages and standard deviations were calculated for apoptosis and colony-forming assays. Unpaired *t*-tests were used to determine the statistical significance by using the online calculator available at the GraphPad Software website (http://www.graphpad.com/quickcalcs/ContMenu.cfm). *P*-values ⩽0.05 were considered to be statistically significant.

## Results

### Physical characteristics of ALM

**Figure 1:**
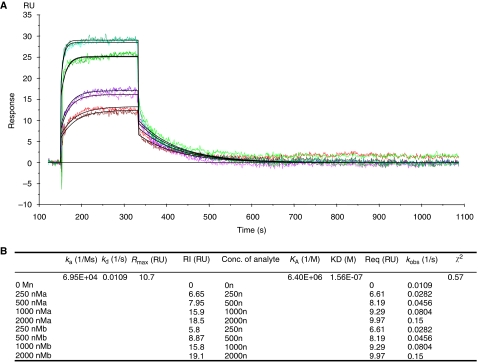
Affinity and binding kinetics of the anti-ErbB3 A5 scFv. *k*_on_ and *k*_off_ rates were determined by surface plasmon resonance and used to determine the binding affinity (*K*_D_) of the A5 scFv. (**A**) Sensorgram fit to 1 : 1 Langmuir binding model. (**B**) Analysis of data.

**Figure 2:**
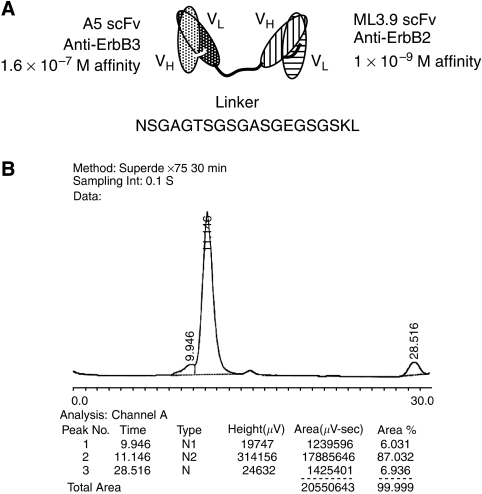
The anti-ErbB2/ErbB3 bs-scFv ALM. (**A**) Cartoon of ALM depicting scFv orientation, linker sequence and kinetic constants of ALM for each target antigen. (**B**) UV adsorption spectrum chromatograph of ALM over Superdex 75 size-exclusion column.

To test the hypothesis that bispecific targeting will increase tumour-targeting selectivity of antibody-based agents, we took advantage of two previously identified scFv, the anti-ErbB2 ML3.9 scFv (Schier *et al*, 1996) and the anti-ErbB3 A5 scFv (Horak *et al*, 2005). The ML3.9 SeFV binds to the ErbB2 ECD with a *K*_D_ of 1.1 × 10^−9^ M (Schier *et al*, 1996) and A5 scFv binds to ErbB3 ECD with a *K*_D_ of 1.6 × 10^−7^ M (Figure 1) as measured by SPR. To create ALM, we fused the genes for the two scFv with an oligonucleotide linker that encodes for a 20 amino acid peptide (Figure 2A). The A5-linker-ML3.9 bs-scFv is soluble when expressed and secreted into the periplasmic space of *E. coli*. Figure 2B depicts a representative chromatograph of ALM over a Superdex 75 column following IMAC purification and demonstrating that the majority (87% in this case) of the protein, as measured by UV absorption, is located in the monomeric peak. Fractions containing the monomeric protein were used for all subsequent analysis. Storage at 4°C for at least 4 weeks does not alter the chromatographic profile of ALM, and protein can be stored at −80°C without an effect on activity as judged by live-cell binding (data not shown). Fusing the scFvs into the ‘ALM’ bs-scFv format did not dramatically alter the antigen-binding kinetics of either scFv. In addition, ALM can bind simultaneously to both ErbB2 and ErbB3 as measured by SPR (data not shown). Although somewhat artificial as compared with binding both antigens on the cell surface, these results indicate that both arms of the bs-scFv were independently active. Together, these data supported further *in vitro* and *in vivo* evaluation of the potential for bivalent binding to enhance targeting selectivity. Further evaluation was also supported by stability of the bs-scFv in human serum. The A5-linker-ML3.9 bs-scFv retained 92 and 89% of its ability to bind to ErbB2‘+’/ErbB3‘+’ SK-OV-3 cells after 48 and 72 h, respectively, when incubated at 37°C in human serum – a time frame compatible with the clearance properties of the antibody. By comparison, trastuzumab retained ∼100% of its activity at 48 h. Consistent with the known biology of ErbB2 and ErbB3, ALM is internalised upon binding to ErbB2‘+’/ErbB3‘+’ cells. Within 15 min, 23±0.85% of ALM was internalised, and by 120 min, 36±0.24% of ALM was internalised with an additional 47±0.16% stably bound to the cell surface.

### The A5-linker-ML3.9 bs-scFv selectively targets ErbB2‘+’/ErbB3‘+’ cells *in vitro*

The ability of bivalent association of ALM to promote selective tumour *vs* normal tissue uptake was addressed using *in vitro*, flow cytometry-based assays. First, MVM2 cells and MDA-MB-468 were used to approximate normal tissues that express only ErbB2 or only ErbB3, respectively, and BT-474 cells represented ErbB2‘+’/ErbB3‘+’-positive tumours. The A5-linker-ML3.9 bs-scFv failed to discriminate between BT-474 (ErbB2‘+’/ErbB3‘+’) and MVM2 (ErbB2‘+’/ErbB3‘−’) cell lines at concentrations ⩾100 nM (Table 2), consistent with high concentrations of ALM favouring monovalent association with cell lines that overexpress ErbB2. At 10 nM, a concentration approaching the *K*_D_ of the ML3.9 scFv for ErbB2, a 3-fold drop in association with MVM2 (ErbB2‘+’/ErbB3‘−’) cells was seen with only a corresponding 1.2-fold drop in association with the BT-474 cells. This resulted in 2.4 times as many BT-474 (ErbB2‘+’/ErbB3‘+’) cells bound by ALM as compared with MVM2 (ErbB2‘+’/ErbB3‘−’) cells. This difference in binding was more pronounced at lower concentrations of ALM, where 6.5 and 9.7 times as many BT-474 cells, as compared with MVM2 (ErbB2‘+’/ErbB3‘−’) cells, were bound by ALM at 1 nM and 100 pM, respectively. Consistent with the low affinity of A5 for ErbB3, binding to the MDA-MB-468 (ErbB2‘−’/ErbB3‘+’) cell line was not detected at concentrations as high as 1 *μ*M.

**Figure 3:**
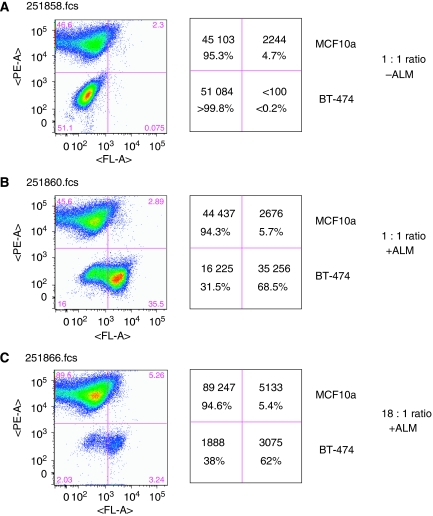
The A5-linker-ML3.9 bs-scFv selectively binds BT-474 tumour cells *in vitro*. Non-labelled BT-474 (ErbB2‘+’/ErbB3‘+’) breast tumour cells were mixed with either an equal (**A** and **B**) or 18-fold excess (**C**) of fluorescently labelled MCF10a (ErbB2‘±’/ErbB3‘±’) normal breast epithelial cells. Cell mixtures were then incubated with buffer (**A**) or 100 nM ALM (**B** and **C**) and binding of ALM to each cell population was determined by flow cytometry with an anti-6XHis tag secondary antibody. MCF10a cells were sorted to the upper quadrants and the non-labelled BT-474 cells were sorted to the lower quadrants. Cells bound by the secondary antibody sorted to the respective right hand quadrants. Images on the left depict the raw flow cytometry data. Values on the right represent the absolute number and overall percentage of each cell type in the respective quadrants.

To model the effects of ErbB2‘+’/ErbB3‘+’ normal tissue on ALM targeting, we used fluorescently labelled MCF10a normal breast epithelial cells mixed with unlabelled BT-474 tumour cells. MCF10a cells express significantly lower levels of both target antigens than BT-474 cells (Table 1). MCF10a (ErbB2‘±’/ErbB3‘±’) and BT-474 (ErbB2‘+’/ErbB3‘+’) cells were readily distinguishable by flow cytometry when mixed at both 1 : 1 and 18 : 1 ratios of MCF10a:BT-474 (Figure 3). The A5-linker-ML3.9 bs-scFv (100 nM) specifically bound to BT-474 cells as compared with the normal MCF10a cells when added to a 1 : 1 mixture of cells. The A5-linker-ML3.9 bs-scFv was detected in 68.5% of the BT-474 cells, whereas MCF10a (ErbB2‘±’/ErbB3‘±’) cells failed to exhibit significant ALM binding. However, ErbB2‘+’/ErbB3‘+’ normal cells are predicted to be in vast excess compared with tumour cells *in vivo*. To begin to mimic this situation, we incubated ALM with BT-474 cells that were mixed with increasing numbers of MCF10a (ErbB2‘±’/ErbB3‘±’) cells. At MCF10a : BT-474 ratios as high as 18 : 1 (maximum ratio tested due to constraints of the assay), the presence of ‘normal tissue’ had no significant impact on the ability of ALM to specifically bind to tumour cells. The A5-linker-ML3.9 bs-scFv was detected on 62% of BT-474 (ErbB2‘+’/ErbB3‘+’) cells present in the assay without significantly binding to MCF10a (ErbB2‘±’/ErbB3‘±’) cells despite the 18-fold higher number of these cells (Figure 3C). This concentration of ALM was capable of binding monovalently to MVM2 cells in such as way as to prevent selective binding between BT-474 (ErbB2‘+’/ErbB3‘+’) and MVM2 (ErbB2‘+’/ErbB3‘−’) cells (Table 2). MVM2 (ErbB2‘+’/ErbB3‘−’) cells express 3 × 10^5^ copies of ErbB2 on the cell surface. This level of expression is equivalent to, or higher than, many ErbB2-positive breast cancer cells lines, such as MDA-361/DYT2 (3.7 × 10^5^), T-47D (5.2 × 10^4^) and MDA-MB-231 (2.8 × 10^4^) when measured *in vitro* by quantitative flow cytometry (Table 1 and Tang *et al*, 2007). Together, these data show that ALM is capable of selectively targeting tumour cells that express high concentrations of both target antigens over cells that express high levels of only one target antigen or low levels of both target antigens. In the latter case, this occurred even when the ErbB2‘+’/ErbB3‘+’ tumour cells represented a minority of the total cells in the assay. This suggests that ALM could exhibit selective tumour-targeting advantages in the clinical setting where both target antigens are present at lower concentrations in a number of normal tissues.

### The A5-linker-ML3.9 bs-scFv specifically targets ErbB2‘+’/ErbB3‘+’ tumours *in vivo*

**Figure 4:**
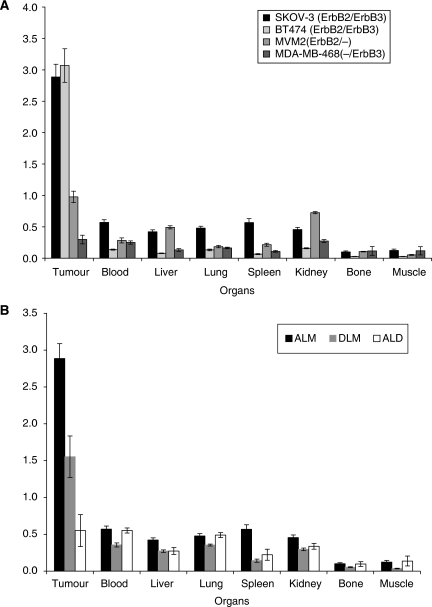
Bispecific binding is required for optimal tumour targeting of the ALM bs-scFv *in vivo*. The biodistributions of radioiodinated ALM, ALD and DLM bs-scFv were analysed 24 h post-injection into xenograft-bearing SCID mice (*n*=5 per cohort). (**A**) Co-expression of ErbB2 and ErbB3 by the targeted tumour is required for optimal targeting of ALM *in vivo*. ^125^I-ALM targeted ErbB2+/ErbB3+ tumour xenografts to ⩾3-fold higher levels than xenografts that express only one of the target antigens. (**B**) Radioiodinated ALM (^125^I-ALM), which is capable of bivalent association with the surface of Sk-OV-3 tumour cells, exhibited increased targeting as compared with ALD and DLM that targeted the tumours monovalently. Error bars represent the standard error of the mean (s.e.m.).

Consistent with the proposed hypothesis that bivalent binding will increase tumour retention, optimal targeting of ALM *in vivo* requires expression of both ErbB2 and ErbB3 on the tumour cell surface (Figure 4A). Radioiodinated ALM (^125^I-ALM) accumulated to similar levels (*P*=0.59) in two different ErbB2‘+’/ErbB3‘+’ tumour xenografts; ^125^I-ALM accumulated in BT-474 and SK-OV-3 tumour xenografts at 2.89±0.2 and 3.07±0.27% injected dose per gram of tissue (% ID per g), respectively, by 24 h post-injection. These levels are statistically greater than those seen in blood (*P*<0.001) or any other tissues analysed (*P*<0.001) and are consistent with the rapid targeting selectivity exhibited by anti-ErbB2 dimeric scFvs against SK-OV-3 xenografts (Adams *et al*, 2006). Both SK-OV-3 and BT-474 cells express low levels of ErbB3, as compared with ErbB2 (Table 1). However, SK-OV-3 cells express levels essentially equal to that of the normal MCF10a breast cell line, and 10-fold lower than BT-474 cells. This difference in ErbB3 expression strongly suggests that effective tumour targeting with bs-scFvs may not require overexpression of both target antigens relative to normal tissue. Rather, monovalent association with an overexpressed antigen may be used to drive initial targeting, with tumour retention being enhanced by avid binding to the second antigen. Consistent with such a model, expression of both antigens, and therefore the potential for divalent binding, is necessary for optimal targeting of ALM. Despite the fact that ALM was retained in the ErbB2‘+’/ErbB3‘−’ MVM2 tumours at statistically greater levels than seen in blood (0.98±0.09 *vs* 0.28±0.04 %ID/g; *P*<0.001), the levels of targeting were significantly lower than those seen in either the BT-474 or SK-OV-3 xenograft models (*P*<0.001). Despite the expression of ErbB3 on the ErbB2‘−’/ErbB3‘+’ MDA-MB-468 tumour model, ^125^I-ALM failed to accumulate to levels above that seen in blood (0.29±0.07 *vs* 0.29±0.03% ID per g; *P*=0.55). The inability to target MDA-MB-468 tumours is consistent with the results of Adams *et al* (1998), which demonstrated that effective targeting and retention of anti-ErbB2 scFv antibodies to SK-OV-3 tumours, a tumour line that expresses 1 × 10^6^ copies of ErbB2 per cell, require a threshold affinity of 1 × 10^−8^ M. The low affinity of the A5 scFv, coupled with the relatively low level of ErbB3 expression (Table 1), suggests that targeting of ALM to ErbB2‘−’/ErbB3‘+’ tissues would be minimal.

Undoubtedly, ErbB2 levels play a significant role in ALM targeting and may explain, at least in part, the lower level of targeting exhibited against MVM2 tumours, as compared with either SK-OV-3 or BT-474 tumours. To control for effects of antigen expression on overall targeting, we constructed bs-scFvs in which either the A5 scFv or the ML3.9 scFv were replaced with a ‘dead’ (D) scFv specific for a non-natural epitope. Targeting of these bs-scFvs was then analysed in a single SK-OV-3 (ErbB2‘+’/ErbB3‘+’) xenograft model. Targeting of ^125^I-DLM through the anti-ErbB2 ML3.9 scFv resulted in 1.55±0.28% ID per g at 24 h post-injection (Figure 4B), significantly more uptake than seen in any non-target tissue (*P*<0.001) but lower than that seen with ALM (*P*<0.002). This level of targeting is similar to that displayed by the isolated ML3.9 scFv (Adams *et al*, 1998), consistent with monovalent targeting by DLM. These data again support the requirement for bivalent association of the bs-scFv with the cell surface to promote optimal targeting. The A5-linker-ML3.9 bs-scFv targeting of MVM2 (ErbB2‘+’/ErbB3‘−’) xenografts and DLM targeting of SK-OV-3 (ErbB2‘+’/ErbB3‘+’) xenografts, both driven solely by ErbB2 expression, showed similar levels of uptake (*P*=0.089), although the trend favored DLM. This trend may again reflect the greater level of ErbB2 expression on the SK-OV-3 (ErbB2‘+’/ErbB3‘+’) cell line. Analogous to the lack of ALM targeting seen against MDA-MB-468 (ErbB2‘−’/ErbB3‘+’) xenografts, ^125^I-ALD, which has a functional anti-ErbB3 arm but no ability to bind to ErbB2, only accumulated to 0.55±0.16% ID per g in SK-OV-3 (ErbB2‘+’/ErbB3‘+’) tumours, a level that did not differ from that seen in blood (*P*=0.78, Figure 4B). This is again consistent with the threshold affinity required for effective *in vivo* targeting described by Adams *et al* (1998). The overall variations in non-target tissue uptake displayed by these bs-scFvs (Figure 4) may be explained, at least in part, by the process of internalization/degradation and release of free iodine; all three constructs displayed the highest degree of variation (s.e.m.=19–43% of total uptake) in stomach uptake, a tissue that expresses the Na/I symporter.

### The A5-linker-ML3.9 bs-scFv displays intrinsic anti-tumour cell activity

**Figure 5:**
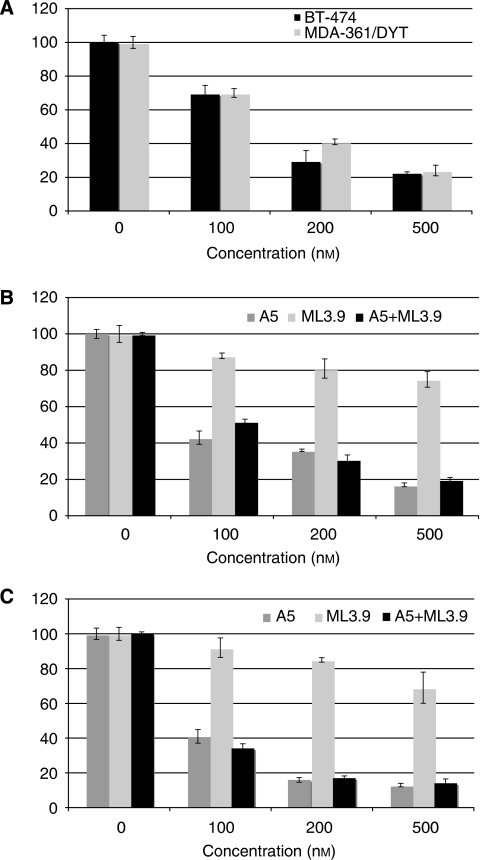
The A5-linker-ML3.9 bs-scFv has intrinsic anti-tumour cell activity. (**A**) Treatment of BT-474 and MDA-361/DYT2 cells with ALM inhibits colony formation in clonogenicity assays. Treatment of (**B**) BT-474 or (**C**) MDA-361/DYT2 cells with A5 scFv, ML3.9 scFv or the combination of both indicates that the majority of the intrinsic anti-tumour cell activity of ALM is due to the anti-ErbB3 A5 scFv arm. Colonies larger than 0.35 mM were counted using an automatic colony counter. Error bars represent the standard deviation.

We hypothesised that targeting the ErbB2/ErbB3 heterodimer with ALM would have the potential to disrupt downstream signalling events. Consistent with this hypothesis, treatment of the ErbB2‘+’/ErbB3‘+’ BT-474 and MDA-361/DYT2 cells with 250 nM ALM induced apoptosis to a level of ∼20% as measured by flow cytometry with Annexin V and 7-AAD, as markers for early and late apoptosis, respectively (data not shown). Although the level of apoptosis is modest compared with the >95% seen in doxorubicin-treated (4 *μ*M) controls, it is greater than the level seen in non-treated cells and cells treated with an irrelevant control scFv (*P*<0.001). The low level of apoptosis is similar to that seen with trastuzumab in certain model systems (Pegram *et al*, 1999). More dramatically, ALM treatment inhibits growth of BT-474 and MDA-361/DYT2 cell lines in standard clonogenicity assays. Chronic treatment of BT-474 and MDA-361/DYT2 cells with ⩾50 nM ALM significantly reduced (*P*<0.001) the ability of the cells to form colonies in a dose-dependent manner as compared with vehicle-treated cells (Figure 5A). Treatment with the clinically relevant MAb pertuzumab (300 nM) inhibited the growth of both cell lines to similar levels as seen with 50 nM ALM (data not shown). When taken together, these data suggest that ALM is eliciting a cytostatic, rather than cytotoxic, effect on the tumour cells. Consistent with this interpretation, ALM (250 nM) treatment increased the percentage of MDA-361/DYT2 cells in the G1 compartment of the cell cycle, as compared to cells treated with either rituximab or vehicle alone (Table 3). As predicted by published literature (Lewis *et al*, 1993; Yakes *et al*, 2002), MDA-361/DYT2 cells were unresponsive to trastuzumab treatment in this assay (Table 3).

Interestingly, the therapeutic activity of ALM is almost entirely because of the anti-ErbB3 A5 arm of the bs-scFv. Chronic treatment of both BT-474 (Figure 5B) and MDA-361/DYT2 (Figure 5C) cells with A5 scFv significantly inhibited the growth of the cells in a dose-dependent manner. Although treatment with ML3.9 produced a modest, although statistically significant, dose-dependent growth inhibition, the combination of A5 and ML3.9 scFv inhibited the growth of both cell lines to the same extent as A5 alone at all dose levels tested.

## Discussion

Classically, bsAbs have been developed to recognise both a tumour-associated antigen and a ‘trigger antigen’ present on the surface of an immune effector cell (Adams and Weiner, 2005) with the aim of redirecting the cytotoxic potential of effector cells against a patient's tumour (Weiner *et al*, 1993; Keler *et al*, 1997). Efforts to create such molecules have encompasssed full-length IgG (Weiner *et al*, 1993), chemically fused Fab' fragments (Shalaby *et al*, 1992; Keler *et al*, 1997) and, more recently, small engineered antibody fragments such as the scFv-based molecules (Shahied *et al*, 2004; Molhoj *et al*, 2007). Preclinical evaluation of these classes of molecules have demonstrated that bsAbs are capable of eliciting effector function against HER2-expressing cell lines *in vitro* and in animal models. In the case of the anti-HER2/Fc*γ*RIII 2B1 bsAb (Weiner *et al*, 1993) and the anti-HER2/Fc*γ*RI MDX-H210 bs-Fab, the efficacy in animal models resulted in phase I clinical trials (Valone *et al*, 1995; Weiner *et al*, 1995).

Unlike the bsAbs mentioned above, the ALM bs-scFv antibody that we describe here binds two distinct tumour-associated antigens. We hypothesised that the effect of avid binding on the targeting of small engineered antibodies, as described by Adams *et al* (2006), could be exploited to increase targeting selectivity. Viewed in this context, our data suggest that bispecific binding of ALM is in fact a major component driving selective targeting of ALM. In particular, this hypothesis is supported by the *in vitro* targeting experiments showing that ALM is capable of selectively binding ErbB2‘+’/ErbB3‘+’ tumour cells when present in a milieu of cells that express either elevated levels of one or normal levels of both target antigens. Although the anti-ErbB2 scFv ML3.9 (*K*_D_=1 × 10^−9^) was sufficient to promote binding of ALM to MVM2 cells (ErbB2‘+’/ErbB3‘−’) at ALM concentrations above 10 nM, it should be noted that MVM2 cells express levels of ErbB2 equal to, or greater than, a number of ErbB2-positive breast cancer cells lines such as MDA-361/DYT2, T-47D and MDA-MB-231 when measured *in vitro* by quantitative flow cytometry (Table 1). The high level of ErbB2 on MVM2 suggests that the levels of selectivity obtained may be an underestimate of those obtainable *in vivo*. This argument is further supported by the selective targeting of BT-474 cells in a background of excess MCF10a cells. An alternative possibility, which is not mutually exclusive with the above hypothesis, is that the anti-ErbB2 scFv ML3.9 is driving initial ALM targeting and that bispecific binding is promoting selective retention of ALM. Such a model supports a hypothesis that the use of moderate- to low-affinity arms in the bs-scFv could significantly limit targeting of tissues that only express one of the tumour antigens, particularly when the antigen is expressed at relatively low levels. Using affinity mutants of the ML3.9 scFv, Adams *et al* (1998) have shown that a *K*_D_ between 10^−7^ and 10^−8^ is necessary to achieve monovalent targeting *in vivo* when the antigen is highly overexpressed. In an effort to optimise the targeting and therapeutic efficacy of ALM, we hypothesise that strategies to alter the affinity for either one or both of the arms for their target antigens can be used to increase the window of selective targeting achievable with bs-scFvs targeting ErbB2‘+’/ErbB3‘+’ tumours although not impairing the overall tumour retention. We believe that the targeting selectivity exhibited by ALM, coupled with its intrinsic anti-tumour cell activity and rapid systemic clearance, makes it a potent therapeutic agent in its own right, as well as an excellent vehicle for the delivery of toxic payloads, such as chemotherapy and radionucleotides. Experiments are underway to test this hypothesis.

Targeting two tumour-associated antigens as a mechanism for modulating signalling within the tumour cell to elicit a therapeutic effect was validated by Zu and colleagues using a single-gene bs-diabody molecule specific for VEGFR2 (KDR) and VEGFR3 (Flt-4). This bs-diabody is capable of binding to both target receptors and blocking ligand-dependent signalling and cell migration *in vitro* (Jimenez *et al*, 2005). The group has also elaborated the bs-diabody format to create a di-diabody, a tetravalent IgG-like structure with an intact Fc-domain. A di-diabody based on the Vh and Vl domains of the anti-EGFR MAb IMC-11F8 and the anti-IGFR MAb IMC- 1121 binds to both IGFR and EGFR. Similar to the parent IgG, the di-diabody blocks ligand-dependent signalling elicited by both EGF and IGF stimulation and promotes an ADCC response when assayed *in vitro*. When injected into mice harbouring IGFR‘+’/EGFR‘+’ HT-29 tumour xenografts, the di-diabody elicits an anti-tumour response that was statistically similar to that seen in mice treated with a combination of both parent antibodies (Lu *et al*, 2005). Elaboration of ALM to include an intact Fc domain, similar to the di-diabody, may be useful for future derivatives of ALM to promote prolonged blood retention and greater tumour uptake, while also providing the potential for ADCC. Alternatively, strategies such as fusion to albumin-binding peptides (Dennis *et al*, 2002; Nguyen *et al*, 2006) may be sufficient to increase blood retention, while retaining the tumour penetration properties of smaller antibody fragments (Adams and Weiner, 2005).

In contrast to the di-diabody described above, ALM targets a heterodimeric pair of RTKs from a unique signal transduction pathway. Misappropriate signalling through the ErbB network promotes processes such as cell survival and is recognised as directly contributing to formation and progression of a number of commonly occurring cancers (Harari and Yarden, 2000; Hynes and Lane, 2005). Unregulated signalling is often a consequence of receptor overexpression. In breast cancer driven by gene amplification and overexpression of ErbB2, disruption of ErbB2-dependent signalling with either trastuzumab or the small-molecule tyrosine kinase inhibitor lapatinib is correlated with clinical efficacy. The ErbB3 growth factor receptor is being increasingly recognised as a central player in ErbB2-driven breast cancer. Immunohistochemical studies have shown that ErbB3 is expressed or overexpressed in greater than 50% of DCIS and invasive breast cancers (Gasparini *et al*, 1994; Bobrow *et al*, 1997; Naidu *et al*, 1998) and correlates with poor prognosis (Rajkumar *et al*, 1996; Lee *et al*, 2002; Bieche *et al*, 2003; Witton *et al*, 2003; Tovey *et al*, 2004). As stated above, the ErbB2 and ErbB3 receptors are frequently co-expressed in human breast cancer, but potentially more indicative of the importance of ErbB2/ErbB3 heterodimers in driving breast cancer progression is the link between escape from ErbB2-targeted therapies and a gain in ErbB3 activity or expression. In patients, high levels of ErbB3 expression predict early escape from trastuzumab therapy (Smith *et al*, 2004), and escape of at least six different breast cancer cell lines from small-molecule TKIs *in vitro* correlates with activation of ErbB3 and concomitant signalling through the Akt pathway (Sergina *et al*, 2007). In addition, recent evidence from Lee-Hoeflich *et al* (2008) demonstrated that ErbB3 was preferentially phosphorylated in ErbB2-amplified breast cancer lines and that knockdown of ErbB3 inhibited growth *in vitro* and *in vivo*. The authors postulate that the addition of pertuzumab, an anti-ErbB2 Mab that blocks ligand-induced ErbB2/ErbB3 dimerisation, to trastuzumab regimens followed at present may provide additional therapeutic benefit by inhibiting ErbB3-dependent signalling. The A5-linker-ML3.9 bs-scFv, by co-targeting this receptor pair, may be capable of a similar activity.

A number of studies strongly suggest that cross talk between ErbB2/ErbB3 heterodimer and ER receptor are responsible for development of tamoxifen resistance (Jhabvala-Romero *et al*, 2003; Stoica *et al*, 2003) and that downregulation of ErbB3 abrogates tamoxifen resistance (Liu *et al*, 2007). Similar studies have linked the ErbB2/ErbB3 heterodimer to the development of androgen-independent prostate cancer (Mellinghoff *et al*, 2004; Ghosh *et al*, 2005; Gregory *et al*, 2005; Edwards *et al*, 2006). Evidence from lung cancer (Engelman *et al*, 2007), pancreatic cancer (Frolov *et al*, 2007), melanoma (Reschke *et al*, 2008) and head and neck cancer (Erjala *et al*, 2006) suggest that ErbB3-dependent signalling mechanisms are important for not only disease progression, but also resistance to EGFR-targeted therapies. Together, these data support a key role for ErbB3 multiple types of cancer and suggest that effective inhibition of ErbB3 may be important for gaining complete therapeutic efficacy with ErbB inhibitors.

The anti-ErbB3 A5 scFv is responsible for the majority of the intrinsic anti-tumour activity of ALM. Despite an affinity for ErbB3 that is unable to promote rapid cell binding in either flow cytometry or *in vivo* targeting studies, we have shown that chronic treatment of ErbB3‘+’ cells with A5 is sufficient to induce growth arrest. Thus, by fusing A5 into the ALM bs-scFv, we have created a vehicle that is capable of effectively targeting this therapeutic scFv to relevant tumour cells. Although the molecular mechanism by which A5 induces its therapeutic effect is not fully elucidated here, one potential mechanism is through blocking ligand-induced heterodimerization of ErbB3. Consistent with such a mechanism is the fact that both pertuzumab, which inhibits ErbB2/ErbB3 heterodimerization and signalling, and ALM are active preclinically against breast cancer cells that are not gene-amplified for ErbB2. This is in direct contrast to the FDA-approved MAb trastuzumab, which requires ErbB2 gene amplification.

In conclusion, we have created a bs-scFv molecule that is capable of mediating selective *in vitro* and *in vivo* tumour targeting and provided what we believe to be the first rigorous evidence of enhanced targeting selectivity by a bsAb specific for two tumour-associated antigen (TAA). The consequence of targeting tumour cells with ALM is effective inhibition of tumour cell growth *in vitro*. Taken together, our results suggest that bispecific targeting of ErbB2 and ErbB3 with ALM may be efficacious *in vivo* against breast cancers with a wider range of ErbB2 expression than those amenable to the trastuzumab therapy available at present. We are addressing this question at present. Moreover, these data also suggest that targeting strategies that depend upon co-expression of two TAAs such as the one used here may increase tumour-targeting specificity and decrease the normal tissue toxicities that are sometimes associated with antibody-based targeting of growth factor receptors. Furthermore, the clearance kinetics of ALM, coupled with its targeting selectivity, may make it an excellent candidate for use as an immunoconjugate for the delivery of cytotoxic agents.

## Figures and Tables

**Table 1:** Expression levels of ErbB2 and ErbB3 on cell lines

**Cell line**	**ErbB2**	**ErbB3**
SK-OV-3	9.6 × 10^5^	3.0 × 10^3^
BT-474	7.6 × 10^5^	5.1 × 10^4^
MDA-361/DYT2	2.9 × 10^5^	2.2 × 10^4^
MDA-MB-468	<1 × 10^2^	1.6 × 10^4^
MCF10a	3.5 × 10^4^	5.0 × 10^3^
T-47D	5.2 × 10^4^	ND
MDA-MB-231	2.8 × 10^4^	ND
MVM2	3.5 × 10^5^	NA

NA=not applicable, cell line is of murine origin and not expected to express human ErbB3; ND=not tested.

Expression levels were determined by quantitative flow cytometry on the basis of the assumption that each antibody bound to two receptors.

**Table 2:** ALM selectively targets ErbB2/ErbB3 positive cells *in vitro*

	**% Positive cells^a^**		
	**BT-474**	**MVM2**	**MDA-MB-468**
**ALM**	**(ErbB2/ErbB3)**	**(ErbB2/−)**	**(−/ErbB3)**
1 μM	99.6	99.2	ND
100 nM	98.6	95.5	ND
10 nM	78.2	32.4	ND
1 nM	23.5	3.6	ND
100 pM	17.0	1.74	ND
Anti-6xHis MAb alone	1.26	1.24	NA

NA=not applicable; ND=not detectable.

a Numbers represent the percentage of each cell type bound by increasing concentrations of ALM after the three lines were stained with CellTracker dyes, mixed together in equal numbers, incubated with ALM and subjected to flow cytometry.

**Table 3:** The A5-linker-ML3.9 bs-scFv arrests MDA-361/DYT2 cells in the G1 phase of the cell cycle

	**% Cells in each cell cycle compartment^a^**		
**Treatment**	**G1**	**S**	**G2**
Non-treated	73.8	16.3	12.9
**The A5-linker-ML3.9 bs-scFv**	**85.3**	**10.4**	**6.7**
Trastuzumab	71.8	18.5	12
Rituximab	74.4	14.1	12.6

a Percentage of cells in each compartment as calculated by the Dean/Jett/Fox algorithm.
